# Effects of Virtual Reality on Anxiety, Stress, Pain, and Patient Satisfaction Among Palestinian Patients Undergoing Colonoscopy: Randomized Controlled Trial

**DOI:** 10.1002/hsr2.72420

**Published:** 2026-04-27

**Authors:** Khulud Mansor, Ibrahim Aqtam, Mustafa Shouli

**Affiliations:** ^1^ Ibn Sina College for Health Professions, Department of Nursing Nablus University for Vocational and Technical Education Nablus Palestine

**Keywords:** anxiety, colonoscopy, pain, Palestine, patient satisfaction, stress, virtual reality

## Abstract

**Background and Aims:**

Colonoscopy frequently causes significant pre‐procedural anxiety and intra‐procedural pain. This randomized controlled trial evaluated immersive, culturally adapted virtual reality (VR) therapy on anxiety, perceived stress, pain, patient satisfaction, and hemodynamic stability in Palestinian adults undergoing colonoscopy.

**Methods:**

In a single‐blinded RCT, 150 patients were randomized to VR (*n* = 75) or standard care (*n* = 75). All received standard pharmacological sedation; the VR group additionally received culturally adapted passive VR distraction. Primary outcomes: State‐Trait Anxiety Inventory (STAI) and Perceived Stress Scale‐10 (PSS‐10). Secondary outcomes: pain (VAS/NRS), satisfaction, and hemodynamic parameters. Intention‐to‐treat analysis was applied.

**Results:**

The VR group showed significantly greater reductions in post‐procedural state‐anxiety (29.7 vs. 38.0; *p* < 0.001; Cohen's *d* = 1.25) and perceived stress (19.1 vs. 23.9; *p* < 0.001; *d* = 0.93). VR patients reported lower pain (VAS 6.4 vs. 8.6; *p* < 0.001; *d* = 1.39) and higher satisfaction (84.9 vs. 63.2; *p* < 0.001; *d* = 1.70). Heart rates were significantly lower at 30 and 45 min intra‐operatively (*p* < 0.001 and *p* = 0.002). VR‐related side effects were minimal (4.0% mild transient dizziness).

**Conclusion:**

Immersive, culturally adapted VR is a highly effective, safe adjunct that significantly reduces anxiety, stress, and pain while improving satisfaction and hemodynamic stability during colonoscopy. Healthcare institutions should consider integrating VR into routine endoscopic care.

**Relevance to Clinical Practice:**

VR is a cost‐effective, patient‐centered strategy that may reduce sedation needs and enhance colorectal cancer screening adherence.

**Trial Registration:** ClinicalTrials.gov identifier: NCT07145203 (registered 21 August 2025).

**Abbreviations**
ASA: American Society of Anesthesiologists
bpm: beats per minute
DBP: diastolic blood pressure
EEG: electroencephalography
HR: heart rate
HRV: heart rate variability
ITT: intention‐to‐treat
MAP: mean arterial pressure
MCID: minimal clinically important difference
NRS: numerical rating scale
PSS‐10: perceived stress scale‐10
RCT: randomized controlled trial
SBP: systolic blood pressure
SD: standard deviation
STAI: state‐trait anxiety inventory
VAS: visual analog scale
VR: virtual reality.

## 1 Introduction

Colonoscopy is the gold standard for colorectal cancer screening and has been shown to reduce associated mortality by up to 68% when applied within organized screening programs [1, 2]. Despite its life‐saving utility, 60%–80% of patients experience moderate‐to‐severe pre‐procedural anxiety, which heightens sympathetic tone, increases intraoperative complications, and reduces future screening compliance [3, 4, 5].

Conventional management relies predominantly on pharmacological sedation with midazolam and fentanyl, which carries risks of respiratory depression, prolonged recovery, and increased cost [6, 7, 8]. Non‐pharmacological alternatives are increasingly sought as complements or substitutes for sedation [9, 10, 11]. Virtual reality (VR) leverages attentional competition (distraction theory) and spinal gating mechanisms (Gate Control Theory) to reduce procedural anxiety and pain [12, 13]. Colonoscopy is particularly suited to VR intervention: patients are conscious or lightly sedated, pre‐procedural anxiety rates are high, the procedure duration (20–45 min) accommodates headset use, and the lateral decubitus position does not interfere with headset placement [14, 15].

Despite growing evidence from Western and Asian settings [16, 17, 18, 19, 20, 21, 22, 23, 24, 25], rigorous RCTs in Middle Eastern populations remain sparse [26, 27]. Palestinian patients present unique cultural considerations—including Islamic values, gender‐specific healthcare preferences, and limited prior exposure to digital health technologies—that necessitate culturally adapted interventions [28, 29, 30, 31, 32]. This RCT evaluated the effect of immersive, culturally adapted VR on anxiety, perceived stress, pain, patient satisfaction, and hemodynamic stability in Palestinian adults undergoing elective colonoscopy.

## 2 Methods

### 2.1 Study Design and Setting

An assessor‐blinded RCT was conducted between September 2024 and December 2024 at the Endoscopy Unit of Rafidia Surgical Governmental Hospital, Nablus, Palestine—a major tertiary referral center performing approximately 3000 colonoscopies annually. The trial followed a pretest–posttest design. Rafidia Hospital remained fully operational throughout the entire study period. Ethical approval was granted by the Institutional Review Board (Ref: R‐2024/A/129/N, August 15, 2024) and the Ministry of Health Ethics Committee (Ref: 162/2224/2024, August 22, 2024). Written informed consent was obtained from all participants prior to any study procedures. The study was conducted in accordance with the Declaration of Helsinki and reported following the CONSORT 2010 Statement [33]. The trial was retrospectively registered at ClinicalTrials.gov (NCT07145203).

### 2.2 Participants

Adults aged 18–75 years scheduled for elective, first‐time diagnostic or surveillance colonoscopy with a STAI score > 38 (moderate‐to‐severe anxiety) and Arabic literacy were eligible. Key exclusion criteria included major psychiatric disorder, epilepsy, uncontrolled hypertension (SBP > 160 or DBP > 100 mmHg), ASA physical status class ≥ III, chronic analgesic use, and any inability to engage with the VR equipment. Full inclusion and exclusion criteria are detailed in Supporting Information S2.

### 2.3 Sample Size

A priori power analysis (G*Power v3.1.9.7) determined a minimum of 40 participants per group (*n *= 80 total) to achieve 80% power at *α *= 0.05 (two‐tailed) for a medium‐to‐large effect size (Cohen's *d *= 0.65), based on prior VR distraction literature in procedural contexts [12, 13]. A total of 150 participants (75 per group) were enrolled to increase power to approximately 99%, accommodate multiple outcomes, enable exploratory subgroup analyses, and enhance external validity.

### 2.4 Randomization and Blinding

Block randomization (fixed blocks of 4) was performed using computer‐generated sequences (Random Allocation Software v1.0) with allocation concealment maintained via sequentially numbered, opaque sealed envelopes prepared by an independent researcher uninvolved in recruitment, intervention delivery, or data collection. Outcome assessors (four trained research nurses) were fully blinded to group allocation, collected all data in a separate room, and had no access to randomization records. Statistical analyses were conducted with groups coded as “A” and “B” until preliminary analyses were complete.

### 2.5 Interventions

#### 2.5.1 VR Group

Participants received standard pharmacological sedation plus immersive, culturally adapted passive VR distraction using a Meta Quest 2 headset (1832 × 1920 px/eye; ~90° field of view). Patients selected one of four nature environments and received Palestinian Arabic‐narrated guided meditation throughout the procedure [34]. VR was initiated at scope insertion and continued until scope withdrawal (typically 15–30 min). Full cultural adaptation details—including Arabic‐language narration, Islamic contemplative principles (muraqabah, tafakkur), Palestinian landscape scenes, multi‐stakeholder review, and pilot testing—are detailed in Supporting Information S3.

#### 2.5.2 Control Group

Standard perioperative care with conscious sedation only (midazolam 1–3 mg IV; fentanyl 25–50 mcg IV, titrated per gastroenterologist judgment). No additional distraction techniques were provided. Environmental conditions were standardized across groups (ambient temperature 22°C–24°C, identical lighting, and room configuration). Full standardized protocols are provided in Supporting Information S4.

### 2.6 Outcomes

Primary outcomes were state‐anxiety assessed with the STAI State subscale (20 items; scores 20–80) at pre‐ and post‐procedure [35], and perceived stress assessed with the PSS‐10 (10 items; scores 0–40) post‐procedure [36]. Both Arabic versions have been validated in Middle Eastern populations (STAI Cronbach's *α *= 0.85–0.91; PSS‐10 *α* = 0.74–0.82); internal consistency in this study was 0.88 and 0.81, respectively. Secondary outcomes were procedural pain (VAS 0–10 and NRS 0–10, assessed within 5 min of scope withdrawal) [37], patient satisfaction (VAS 0–100, assessed 30 min post‐procedure), and hemodynamic parameters (HR, SBP, DBP at baseline; 15, 30, and 45 min intra‐operatively; and post‐procedure). Sedation requirements, procedure duration, and VR‐related adverse events (assessed using the Simulator Sickness Questionnaire [38]) were also documented.

### 2.7 Statistical Analysis

All analyses were conducted on an intention‐to‐treat basis using IBM SPSS v27.0. Between‐group comparisons used independent *t*‐tests or Mann–Whitney U tests for continuous variables and *χ* ^2^ or Fisher's exact tests for categorical variables. Paired *t*‐tests assessed within‐group STAI changes. Hemodynamic data were analyzed using repeated‐measures ANOVA and mixed‐model ANOVA. Effect sizes (Cohen's *d*) were computed for all primary outcomes [39]; MCIDs applied were: STAI 5–7 points, PSS‐10 3–4 points, VAS pain 1.3–1.7 points, and satisfaction 15–20 points [40]. All tests were two‐sided (*α *= 0.05). No Bonferroni correction was applied to primary outcomes given their pre‐specified, orthogonal nature [41]. SAMPL guidelines were followed throughout [42].

## 3 Results

### 3.1 Patient Flow and Baseline Characteristics

Of 500 screened patients, 350 were excluded (280 did not meet the inclusion criteria; 70 declined to participate). A total of 150 patients were enrolled and randomized (VR *n *= 75; control *n *= 75). No post‐randomization dropouts occurred (0% attrition rate). Full screening and enrollment details are presented in the CONSORT flow diagram (Supporting Information S1). Groups were well‐balanced at baseline across all demographic, physiological, and psychological parameters (Table 1).

**Table 1:** Baseline demographic and clinical characteristics (*N *= 150).

Variable	VR group (*n *= 75)	Control group (*n* = 75)	*p* value
Age, years, mean (SD)	48.4 (10.6)	49.7 (11.1)	0.461^a^
Female, *n* (%)	39 (52.0)	38 (50.7)	0.872^b^
Secondary education or higher, *n* (%)	57 (76.0)	54 (72.0)	0.571^b^
Employed, *n* (%)	38 (50.7)	41 (54.7)	0.644^b^
Married, *n* (%)	63 (84.0)	62 (82.7)	0.824^b^
SBP, mmHg, mean (SD)	128.1 (9.9)	127.5 (10.0)	0.701^a^
DBP, mmHg, mean (SD)	78.1 (7.0)	77.8 (6.8)	0.782^a^
Heart rate, bpm, mean (SD)	82.7 (8.5)	83.3 (8.9)	0.668^a^
State‐anxiety (STAI), mean (SD)	41.7 (7.9)	42.2 (7.9)	0.668^a^

Abbreviations: DBP, diastolic blood pressure; SBP, systolic blood pressure; STAI, state‐trait anxiety inventory; VR, virtual reality.

^a^ Independent samples *t*‐test.

^b^ *χ* ^2^ test.

### 3.2 Primary and Secondary Outcomes

The VR group demonstrated large, clinically meaningful improvements across all primary and secondary outcomes (Table 2). Post‐procedural state‐anxiety fell to the low‐anxiety range in the VR group (29.7) versus the moderate‐anxiety range in controls (38.0), representing mean reductions of 12.0 and 4.2 points, respectively—both exceeding the MCID of 5–7 points. The PSS‐10 difference of 4.8 points exceeded the MCID of 3–4 points. Pain reduction was 2.2 points on the VAS (MCID: 1.3–1.7 points), with strong convergent validity between VAS and NRS (*r *= 0.94, *p* < 0.001). Patient satisfaction improved by 21.7 points, exceeding the MCID of 15–20 points. All effect sizes were large (*d *≥ 0.93).

**Table 2:** Primary and secondary outcomes (*N *= 150).

Outcome	VR group mean (SD)	Control group mean (SD)	*p* value	Cohen's *d*
State‐anxiety pre‐procedure (STAI)	41.7 (7.9)	42.2 (7.9)	0.668	0.07
State‐anxiety post‐procedure (STAI)	29.7 (6.2)	38.0 (7.2)	< 0.001^a^	1.25
Change in state‐anxiety	−12.0 (6.8)	−4.2 (5.9)	< 0.001^a^	1.22
Perceived stress PSS‐10 (post)	19.1 (4.9)	23.9 (5.4)	< 0.001^a^	0.93
Pain VAS (0–10)	6.4 (1.8)	8.6 (1.4)	< 0.001^a^	1.39
Pain NRS (0–10)	6.3 (1.7)	8.5 (1.5)	< 0.001^a^	1.38
Satisfaction VAS (0–100)	84.9 (11.2)	63.2 (14.3)	< 0.001^a^	1.70

Abbreviations: NRS, numerical rating scale; PSS‐10, perceived stress scale‐10; VAS, visual analog scale; VR, virtual reality.

^a^ Statistically significant (*p *< 0.05); all Cohen's *d* values ≥ 0.8 indicate large effect sizes.

### 3.3 Hemodynamic Parameters

Blood pressure did not differ significantly between groups at any time point. Heart rate showed a progressive between‐group divergence, with significantly lower values in the VR group at 30 and 45 min intra‐operatively, converging at post‐procedure assessment (Table 3). This pattern reflects a cumulative autonomic calming effect of sustained VR immersion [43].

**Table 3:** Heart rate during colonoscopy (bpm, mean [SD]) and blood pressure significance.

Time point	HR VR bpm (SD)	HR control bpm (SD)	*p* value	SBP/DBP significant
Baseline	82.7 (8.5)	83.3 (8.9)	0.668	No
15 min intra‐operative	79.1 (7.8)	81.5 (8.2)	0.067	No
30 min intra‐operative	76.4 (7.1)	80.8 (7.9)	< 0.001^a^	No
45 min intra‐operative	75.8 (6.9)	79.5 (7.5)	0.002^a^	No
Post‐procedure	77.3 (7.3)	78.9 (7.8)	0.187	No

Abbreviations: bpm, beats per minute; VR, virtual reality.

^a^ Statistically significant (*p *< 0.05). SBP and DBP showed no significant between‐group differences at any time point (all *p* > 0.37).

### 3.4 Sedation, Procedural Data, and Adverse Events

Sedation requirements trended lower in the VR group (14.7% vs. 21.3% requiring supplemental doses; relative reduction 31%; *p* = 0.295). Procedure duration and cecal intubation rates were equivalent between groups. VR‐related side effects were minimal, transient, and self‐limiting. No serious adverse events occurred in either group (Table 4).

**Table 4:** Sedation, procedural data, and adverse events (*N *= 150).

Variable	VR group (*n *= 75)	Control group (*n *= 75)	*p* value
Sedation and procedural data			
Required additional sedation, *n* (%)	11 (14.7)	16 (21.3)	0.295^a^
Total midazolam dose, mg, mean (SD)	2.1 (0.8)	2.3 (0.9)	0.149^b^
Total fentanyl dose, mcg, mean (SD)	38.2 (12.4)	41.7 (13.8)	0.092^b^
Total procedure time, min, mean (SD)	28.7 (8.3)	29.5 (9.1)	0.553^b^
Cecal intubation achieved, *n* (%)	74 (98.7)	73 (97.3)	0.620^c^
Adverse events			
Any VR‐related side effect (mild dizziness), *n* (%)	3 (4.0)	0 (0.0)	0.080^c^
Desaturation (SpO₂ < 90%), *n* (%)	2 (2.7)	3 (4.0)	0.648^a^
Hypotension (MAP < 65 mmHg), *n* (%)	1 (1.3)	2 (2.7)	0.559^c^
Bradycardia (HR < 50 bpm), *n* (%)	0 (0.0)	1 (1.3)	0.316^c^
Serious adverse events	None	None	—

Abbreviations: HR, heart rate; MAP, mean arterial pressure; VR, virtual reality.

^a^ *χ* ^2^ test.

^b^ Independent samples *t*‐test.

^c^ Fisher's exact test.

### 3.5 VR Acceptability

The Tropical Beach scene was most popular (38.7%), followed by Forest Walk (30.7%), Underwater Diving (20.0%), and Snowy Landscape (10.7%). Of VR group participants, 96.0% rated the experience as comfortable or very comfortable, 94.7% would recommend VR to other patients, and 97.3% would choose VR for a future colonoscopy.

## 4 Discussion

This single‐blinded RCT demonstrates that immersive, culturally adapted VR produces large, clinically meaningful improvements in anxiety (*d *= 1.25), perceived stress (*d *= 0.93), pain (*d *= 1.39), and patient satisfaction (*d *= 1.70) in Palestinian patients undergoing colonoscopy. All effect sizes exceeded established MCIDs and surpassed the pooled estimates reported in the broader VR meta‐analytic literature [44].

### 4.1 Mechanistic Basis

The observed pain reduction is consistent with Gate Control Theory [12, 13]: VR's non‐noxious multisensory input activates large‐diameter afferent fibers that competitively inhibit nociceptive signal transmission at the dorsal horn, reducing perceived pain despite identical procedural stimulation. The large effect size (*d *= 1.39) further reflects the role of cognitive load reduction: neuroimaging evidence confirms that immersive VR suppresses activation in pain‐processing regions, including the anterior cingulate cortex, insula, and thalamus [45, 46]. The anxiety reduction aligns with Distraction Theory [47, 48]: the immersive virtual environment monopolizes attentional resources that would otherwise focus on procedural anxiety triggers such as scope awareness, procedural sounds, and anticipatory discomfort. The emotional arousal and stress‐reducing properties of simulated natural environments have been well documented [49, 50, 51]. The progressive heart‐rate divergence—minimal at 15 min but significant at 30 and 45 min—indicates a cumulative autonomic calming effect consistent with Roy's Adaptation Model [52, 53], whereby sustained VR exposure increasingly counteracts mounting procedural stress through parasympathetic activation [54].

### 4.2 Cultural Adaptation and Satisfaction

The exceptionally large satisfaction effect size (*d *= 1.70)—among the highest reported in the VR procedural literature [55, 56]—likely reflects the synergistic impact of cultural resonance, patient agency in environment selection, and technology novelty [57]. Palestinian‐ and Islamic‐adapted content (Arabic narration, Palestinian landscapes, Islamic contemplative practices) aligned with local values in ways that generic VR content could not achieve, enhancing patient engagement, and therapeutic effectiveness [28, 29, 30, 31, 32]. The preference for coastal and forest environments over snow landscapes (10.7%) likely reflects climatological and cultural familiarity, further supporting the value of population‐specific content customization.

### 4.3 Clinical Implications

VR did not interfere with procedural efficiency (duration: 28.7 vs. 29.5 min; *p* = 0.553), confirming its feasibility for routine implementation. The trend toward reduced supplemental sedation (14.7% vs. 21.3%; *p* = 0.295) was not statistically significant, likely reflecting insufficient power for this secondary outcome; a 31% relative reduction is clinically noteworthy and warrants evaluation in dedicated trials. Reduced sedation would lower medication costs, shorten recovery, reduce risks of respiratory depression [58], and expand eligibility to patients with sedation contraindications [59]. Non‐pharmacological distraction techniques such as music have shown similar but smaller effects [60, 61], whereas VR offers superior immersion [62]. Institutions implementing VR should address initial equipment cost (~$700–$900 for two procedure rooms; recoverable within months through sedation savings), a standardized 2‐h staff training protocol, five additional minutes for setup, and structured cultural adaptation protocols for diverse patient populations. Improved patient experience during colonoscopy is critically linked to future adherence to colorectal cancer screening programs [63, 64, 65, 66].

### 4.4 Limitations

Key limitations include: single‐center design restricting generalizability; inability to blind participants or procedural staff to group allocation; retrospective trial registration; perceived stress measured post‐procedure only, precluding a baseline‐to‐follow‐up change analysis; exclusive use of guided meditation VR without comparison to interactive modalities; absence of long‐term follow‐up for screening adherence; and lack of objective neurophysiological measures (HRV, cortisol, EEG) to directly confirm mechanistic pathways [47, 67]. Future multi‐center RCTs should address these gaps and include dose‐response investigations and comparative VR content evaluations.

## 5 Conclusions

Immersive, culturally adapted VR is a safe, effective, and highly acceptable adjunct to standard colonoscopy care, producing large reductions in anxiety, stress, and pain alongside markedly improved patient satisfaction and hemodynamic stability, with minimal side effects and no interference with procedural quality. Healthcare institutions serving diverse populations should consider integrating culturally tailored VR as a standard adjunctive therapy in endoscopy units. Future research should prioritize mechanistic characterization, multi‐center generalizability, sedation‐reduction protocols, and long‐term screening adherence outcomes.

## Author Contributions

**Khulud Mansor:** conceptualization, data curation, formal analysis, original draft, cultural adaptation coordination. **Ibrahim Aqtam:** methodology, theoretical framework, supervision, review, and editing. **Mustafa Shouli:** statistical oversight, validation, supervision, review, and editing. All authors approved the final manuscript.

## Funding

The authors have nothing to report.

## Ethics Statement

This study was approved by the Institutional Review Board (R‐2024/A/129/N) and the Ministry of Health Ethics Committee (162/2224/2024). The study was conducted in accordance with the Declaration of Helsinki.

## Consent

Written informed consent was obtained from all participants prior to enrollment.

## Conflicts of Interest

The authors declare no conflicts of interest.

## What Does This Paper Contribute to the Wider Global Community?

This study provides robust, cross‐cultural evidence that immersive VR is a powerful and safe adjunct to standard care for managing procedural anxiety, stress, and pain in adult patients undergoing colonoscopy.
It demonstrates that comprehensive cultural adaptation of digital health interventions, encompassing language, visual content, religious principles, and community stakeholder review, is both feasible and functionally important for optimizing therapeutic effectiveness in diverse global settings.
The findings offer a practical, scalable implementation model for patient‐centered, resource‐conscious healthcare systems seeking to improve the procedural experience and potentially reduce pharmacological sedation requirements.

## Transparency Statement

The lead author, Khulud Mansor, affirms that this manuscript is an honest, accurate, and transparent account of the study being reported; that no important aspects of the study have been omitted; and that any discrepancies from the study as planned (and, if relevant, registered) have been explained.

## Supporting information

Supporting File 1

Supporting File 2

Supporting File 3

Supporting File 4

## Data Availability

Data are available on request from the corresponding author, subject to privacy and ethical restrictions.

## References

[1] M. Bretthauer , M. Løberg , P. Wieszczy , et al., “Effect of Colonoscopy Screening on Risks of Colorectal Cancer and Related Death,” New England Journal of Medicine 387, no. 17 (2022): 1547–1556. 36214590 10.1056/NEJMoa2208375

[2] A. Shaukat , S. J. Mongin , M. S. Geisser , et al., “Long‐Term Mortality After Screening for Colorectal Cancer,” New England Journal of Medicine 369, no. 12 (2013): 1106–1114. 24047060 10.1056/NEJMoa1300720

[3] R. A. Sheikh , T. P. Prindiville , and D. Arenson , “Does the Variable in the Type of Sedation During Colonoscopy Affect the Quality of Examination?,” Gastrointestinal Endoscopy 60, no. 3 (2004): 397–403.15332030

[4] A. Luck , S. Pearson , G. Maddem , and P. Hewett , “Effects of Video Information on Precolonoscopy Anxiety and Knowledge: A Randomised Trial,” Lancet 354, no. 9195 (1999): 2032–2035. 10636368 10.1016/s0140-6736(98)10495-6

[5] Y. Takahashi , H. Tanaka , M. Kinjo , and K. Sakumoto , “Prospective Evaluation of Factors Predicting Difficulty and Pain During Sedation‐Free Colonoscopy,” Diseases of the Colon & Rectum 48, no. 7 (2005): 1295–1300. 15793639 10.1007/s10350-004-0940-1

[6] F. Radaelli , G. Meucci , G. Sgroi , and G. Minoli , “Technical Performance of Colonoscopy: The Key Role of Sedation/Analgesia,” American Journal of Gastroenterology 103, no. 5 (2008): 1122–1130. 18445096 10.1111/j.1572-0241.2007.01778.x

[7] L. B. Cohen , M. H. DeLegge , J. Aisenberg , et al., “AGA Institute Review of Endoscopic Sedation,” Gastroenterology 133, no. 2 (2007): 675–701. 17681185 10.1053/j.gastro.2007.06.002

[8] D. S. Early , J. R. Lightdale , J. J. Vargo , et al., “Guidelines for Sedation and Anesthesia in GI Endoscopy,” Gastrointestinal Endoscopy 87, no. 2 (2018): 327–337. 29306520 10.1016/j.gie.2017.07.018

[9] R. Wang , X. Huang , Y. Wang , and M. Akbari , “Non‐Pharmacologic Approaches in Preoperative Anxiety, a Comprehensive Review,” Frontiers in Public Health 10 (2022): 854673. 35480569 10.3389/fpubh.2022.854673 PMC9035831

[10] I. Lanini , T. Amass , C. S. Calabrisotto , et al., “The Influence of Psychological Interventions on Surgical Outcomes,” Journal of Anesthesia, Analgesia and Critical Care 2, no. 1 (2022): 31. 37386591 10.1186/s44158-022-00057-4 PMC10245433

[11] K. L. Kwekkeboom and E. Gretarsdottir , “Systematic Review of Relaxation Interventions for Pain,” Journal of Nursing Scholarship 38, no. 3 (2006): 269–277. 17044345 10.1111/j.1547-5069.2006.00113.x

[12] P. Indovina , D. Barone , L. Gallo , A. Chirico , G. De Pietro , and A. Giordano , “Virtual Reality as a Distraction Intervention to Relieve Pain and Distress During Medical Procedures: A Comprehensive Literature Review,” Clinical Journal of Pain 34, no. 9 (2018): 858–877. 29485536 10.1097/AJP.0000000000000599

[13] B. Mallari , E. K. Spaeth , and H. Goh , “Virtual Reality as an Analgesic for Acute and Chronic Pain in Adults: A Systematic Review and Meta‐Analysis,” Journal of Pain Research 12 (2019): 2053–2085. 31308733 10.2147/JPR.S200498 PMC6613199

[14] P. L. Chiu , H. Li , K. Y. L. Yap , K. C. Lam , P. R. Yip , and C. L. Wong , “Virtual Reality‐Based Intervention to Reduce Preoperative Anxiety in Adults Undergoing Elective Surgery: A Randomized Clinical Trial,” JAMA Network Open 6, no. 10 (2023): e2340588. 37906193 10.1001/jamanetworkopen.2023.40588 PMC10618840

[15] S. P. Cohen , T. L. Doshi , C. S. Munjupong , et al., “Multicenter, Randomized, Controlled Comparative‐Effectiveness Study Comparing Virtual Reality to Sedation and Standard Local Anesthetic for Pain and Anxiety During Epidural Steroid Injections,” Lancet Regional Health—Southeast Asia 27 (2024): 100437. 39036653 10.1016/j.lansea.2024.100437 PMC11259926

[16] H. G. Hoffman , G. T. Chambers , W. J. Meyer , et al., “Virtual Reality as an Adjunctive Non‐Pharmacologic Analgesic for Acute Burn Pain During Medical Procedures,” Annals of Behavioral Medicine 41, no. 2 (2011): 183–191. 21264690 10.1007/s12160-010-9248-7 PMC4465767

[17] K. Niki , Y. Okamoto , and I. Maeda , “A Novel Distraction Technique for Control of Burn Pain,” Journal of Burn Care and Research 39, no. 2 (2018): 157–164.

[18] Y. H. Sokmen , S. Ozdemir , L. Kaya , and H. Polat , “The Effect of Virtual Reality Accompanied by Music on Women's Perceived Pain During Episiotomy Repair: A Randomized Controlled Trial,” Journal of Maternal‐Fetal & Neonatal Medicine 37, no. 1 (2024): 2347505.

[19] Z. Kahyaoglu Cakmak , E. Unal , M. Yilmaz , and M. Dincer , “The Effect of Virtual Reality on Fetal Movement, Maternal Satisfaction, and Anxiety During Non‐Stress Test: A Randomized Controlled Trial,” Journal of Perinatal & Neonatal Nursing 38, no. 2 (2024): 170–179.

[20] S. Dayanir and E. Cakir Unal , “The Effect of Virtual Reality Glasses During Pap Smear Tests on Anxiety, Pain, and Satisfaction: A Randomized Controlled Trial,” Journal of Obstetric, Gynecologic, and Neonatal Nursing 53, no. 3 (2024): 291–301.

[21] H. Polat , L. Kaya , S. Ozdemir , and H. Yasin Sokmen , “The Impact of Virtual Reality With Relaxation Music on Pain, Anxiety, and Satisfaction During Intrauterine Device Insertion: A Randomized Controlled Trial,” Journal of Clinical Nursing 33, no. 5 (2024): 1899–1909

[22] R. Eijlers , E. M. W. J. Utens , L. M. Staals , et al., “Systematic Review and Meta‐Analysis of Virtual Reality in Pediatrics: Effects on Pain and Anxiety,” Anesthesia & Analgesia 129, no. 5 (2019): 1344–1353. 31136330 10.1213/ANE.0000000000004165 PMC6791566

[23] S. Riches , P. Jeyarajaguru , L. Taylor , et al., “Virtual Reality Relaxation for People With Mental Health Conditions: A Systematic Review,” Social Psychiatry and Psychiatric Epidemiology 58, no. 7 (2023): 989–1007. 36658261 10.1007/s00127-022-02417-5 PMC9852806

[24] M. Li , Z. Yu , H. Li , et al., “Effects of Virtual Reality Therapy for Patients With Breast Cancer During Chemotherapy: Randomized Controlled Trial,” JMIR Serious Games 12 (2024): e53825. 39417797 10.2196/53825 PMC11500621

[25] E. Zasadzka , A. Pieczyńska , T. Trzmiel , and K. Hojan , “Virtual Reality as a Promising Tool Supporting Oncological Treatment in Breast Cancer,” International Journal of Environmental Research and Public Health 18, no. 16 (2021): 8768. 34444513 10.3390/ijerph18168768 PMC8393836

[26] T. Al‐Ghraiybah , H. Alshraideh , and M. Alhadidi , “Perception of Cancer Pain Management Among Oncology Nurses in Jordan: A Qualitative Study,” Journal of Transcultural Nursing 31, no. 1 (2020): 77–85.

[27] M. A. A. Doumit , H. Abu‐Saad Huijer , and J. H. Kelley , “Implementation of a Palliative Care Program in Lebanon: Challenges and Successes,” Journal of Palliative Care 23, no. 2 (2007): 101–109.

[28] F. G. Castro , M. Barrera , and L. K. Holleran Steiker , “Issues and Challenges in the Design of Culturally Adapted Evidence‐Based Interventions,” Annual Review of Clinical Psychology 6 (2010): 213–239. 10.1146/annurev-clinpsy-033109-132032 PMC4262835 20192800

[29] G. Bernal , M. I. Jiménez‐Chafey , and M. M. Domenech Rodríguez , “Cultural Adaptation of Treatments: A Resource for Considering Culture in Evidence‐Based Practice,” Professional Psychology: Research and Practice 40, no. 4 (2009): 361–368.

[30] R. Giacaman , R. Khatib , L. Shabaneh , et al., “Health Status and Health Services in the Occupied Palestinian Territory,” Lancet 373, no. 9666 (2009): 837–849.

[31] W. Hammoudeh , A. Mataria , L. Wick , and R. Giacaman , “In Search of Health: Quality of Life Among Postpartum Palestinian Women,” Expert Review of Pharmacoeconomics & Outcomes Research 9, no. 2 (2009): 123–132. 19402799 10.1586/erp.09.8

[32] R. Sayigh , Palestinians: From Peasants to Revolutionaries (Zed Books, 2007).

[33] K. F. Schulz , D. G. Altman , and D. Moher , “CONSORT 2010 Statement: Updated Guidelines for Reporting Parallel Group Randomised Trials,” BMJ 340 (2010): c332. 20332509 10.1136/bmj.c332 PMC2844940

[34] A. S. Rizzo and R. Shilling , “Clinical Virtual Reality Tools to Advance the Prevention, Assessment, and Treatment of PTSD,” European Journal of Psychotraumatology 8, no. S5 (2017): 1414560. 29372007 10.1080/20008198.2017.1414560 PMC5774399

[35] C. D. Spielberger , Manual for the State‐Trait Anxiety Inventory (Consulting Psychologists Press, 1983).

[36] S. Cohen , T. Kamarck , and R. Mermelstein , “A Global Measure of Perceived Stress,” Journal of Health and Social Behavior 24, no. 4 (1983): 385–396. 6668417

[37] M. J. Hjermstad , P. M. Fayers , D. F. Haugen , et al., “Studies Comparing Numerical Rating Scales, Verbal Rating Scales, and Visual Analogue Scales for Assessment of Pain Intensity in Adults: A Systematic Literature Review,” Journal of Pain and Symptom Management 41, no. 6 (2011): 1073–1093. 21621130 10.1016/j.jpainsymman.2010.08.016

[38] R. S. Kennedy , N. E. Lane , K. S. Berbaum , and M. G. Lilienthal , “Simulator Sickness Questionnaire: An Enhanced Method for Quantifying Simulator Sickness,” International Journal of Aviation Psychology 3, no. 3 (1993): 203–220.

[39] J. Cohen , Statistical Power Analysis for the Behavioral Sciences, 2nd ed. (Lawrence Erlbaum Associates, 1988).

[40] R. H. Dworkin , D. C. Turk , K. W. Wyrwich , et al., “Interpreting the Clinical Importance of Treatment Outcomes in Chronic Pain Clinical Trials: IMMPACT Recommendations,” Journal of Pain 9, no. 2 (2008): 105–121. 18055266 10.1016/j.jpain.2007.09.005

[41] T. V. Perneger , “What's Wrong With Bonferroni Adjustments,” BMJ 316, no. 7139 (1998): 1236–1238. 9553006 10.1136/bmj.316.7139.1236 PMC1112991

[42] T. A. Lang and D. G. Altman , “Basic Statistical Reporting for Articles Published in Biomedical Journals: The Statistical Analyses and Methods in the Published Literature or the SAMPL Guidelines,” International Journal of Nursing Studies 52, no. 1 (2015): 5–9, 10.1016/j.ijnurstu.2014.09.006. 25441757

[43] A. S. Jansen , X. V. Nguyen , V. Karpitskiy , T. C. Mettenleiter , and A. D. Loewy , “Central Command Neurons of the Sympathetic Nervous System,” Science 270, no. 5236 (1995): 644–646. 7570024 10.1126/science.270.5236.644

[44] J. Dascal , M. Reid , W. W. IsHak , et al., “Virtual Reality and Medical Inpatients: A Systematic Review of Randomized, Controlled Trials,” Innovations in Clinical Neuroscience 14, no. 1–2 (2017): 14–21. 28386517 PMC5373791

[45] H. G. Hoffman , T. L. Richards , B. Coda , et al., “Modulation of Thermal Pain‐Related Brain Activity With Virtual Reality: Evidence From FMRI,” Neuroreport 15, no. 6 (2004): 1245–1248. 15167542 10.1097/01.wnr.0000127826.73576.91

[46] B. K. Wiederhold , K. Gao , C. Sulea , and M. D. Wiederhold , “Virtual Reality as a Distraction Technique in Chronic Pain Patients,” Cyberpsychology, Behavior and Social Networking 17, no. 6 (2014): 346–352. 24892196 10.1089/cyber.2014.0207 PMC4043365

[47] K. D. McCaul and J. M. Malott , “Distraction and Coping With Pain,” Psychological Bulletin 95, no. 3 (1984): 516–533. 6399756

[48] A. Pourmand , S. Davis , A. Marchak , T. Whiteside , and N. Sikka , “Virtual Reality as a Clinical Tool for Pain Management,” Current Pain and Headache Reports 22, no. 12 (2018): 53. 29904806 10.1007/s11916-018-0708-2

[49] A. Felnhofer , O. D. Kothgassner , M. Schmidt , et al., “Is Virtual Reality Emotionally Arousing? Investigating Five Emotion‐Inducing Virtual Park Scenarios,” International Journal of Human‐Computer Studies 82 (2015): 48–56.

[50] M. H. E. M. Browning , K. J. Mimnaugh , C. J. van Riper , H. K. Laurent , and S. M. LaValle , “Can Simulated Nature Support Mental Health? Comparing Short, Single‐Doses of 360‐Degree Nature Videos in Virtual Reality With the Outdoors,” Frontiers in Psychology 10 (2020): 2667. 32010003 10.3389/fpsyg.2019.02667 PMC6974516

[51] J. Diemer , A. Mühlberger , P. Pauli , and P. Zwanzger , “Virtual Reality Exposure in Anxiety Disorders: Impact on Psychophysiological Reactivity,” World Journal of Biological Psychiatry 15, no. 6 (2014): 427–442. 10.3109/15622975.2014.892632 24666248

[52] C. Roy , The Roy Adaptation Model, 3rd ed. (Pearson Education, 2009).

[53] C. Roy and H. A. Andrews , The Roy Adaptation Model, 2nd ed. (Appleton & Lange, 1999).

[54] D. H. Hellhammer , S. Wüst , and B. M. Kudielka , “Salivary Cortisol as a Biomarker in Stress Research,” Psychoneuroendocrinology 34, no. 2 (2009): 163–171. 19095358 10.1016/j.psyneuen.2008.10.026

[55] S. Triberti , C. Repetto , and G. Riva , “Psychological Factors Influencing the Effectiveness of Virtual Reality‐Based Analgesia: A Systematic Review,” Cyberpsychology, Behavior and Social Networking 17, no. 6 (2014): 335–345. 24892195 10.1089/cyber.2014.0054

[56] J. Sitzia and N. Wood , “Patient Satisfaction: A Review of Issues and Concepts,” Social Science & Medicine 45, no. 12 (1997): 1829–1843. 9447632 10.1016/s0277-9536(97)00128-7

[57] A. Li , Z. Montaño , V. J. Chen , and J. I. Gold , “Virtual Reality and Pain Management: Current Trends and Future Directions,” Pain Management 1, no. 2 (2011): 147–157. 21779307 10.2217/pmt.10.15 PMC3138477

[58] D. K. Rex , V. P. Deenadayalu , E. Eid , et al., “Endoscopist‐Directed Administration of Propofol: A Worldwide Safety Experience,” Gastroenterology 137, no. 4 (2009): 1229–1237. 19549528 10.1053/j.gastro.2009.06.042

[59] S. Vijan , J. M. Inadomi , and R. A. Hayward , “Adherence to Colorectal Cancer Screening: A Randomized Clinical Trial of Competing Strategies,” Archives of Internal Medicine 172, no. 7 (2012): 575–582. 22493463 10.1001/archinternmed.2012.332 PMC3360917

[60] D. W. H. Lee , K. W. Chan , C. M. Poon , et al., “Relaxation Music Decreases the Dose of Patient‐Controlled Sedation During Colonoscopy,” Gastrointestinal Endoscopy 55, no. 1 (2002): 33–36. 11756911 10.1067/mge.2002.120387

[61] D. Rudin , A. Kiss , R. Wetz , and V. Sottile , “Music in the Endoscopy Suite: A Meta‐Analysis of Randomized Controlled Studies,” Endoscopy 39, no. 4 (2007): 507–510. 17554644 10.1055/s-2007-966362

[62] I. M. Kopton and P. Kenning , “Near‐Infrared Spectroscopy (NIRS) as a New Tool for Neuroeconomic Research,” Frontiers in Human Neuroscience 8 (2014): 549. 25147517 10.3389/fnhum.2014.00549 PMC4124877

[63] M. J. Sewitch , C. Fournier , A. Ciampi , and A. Dyachenko , “Adherence to Colorectal Cancer Screening Guidelines in Canada,” BMC Gastroenterology 7 (2007): 39. 17910769 10.1186/1471-230X-7-39 PMC2194682

[64] S. Gupta , E. A. Halm , D. C. Rockey , et al., “Comparative Effectiveness of Fecal Immunochemical Test Outreach, Colonoscopy Outreach, and Usual Care for Boosting Colorectal Cancer Screening Among the Underserved: A Randomized Clinical Trial,” JAMA Internal Medicine 173, no. 18 (2013): 1725–1732. 23921906 10.1001/jamainternmed.2013.9294 PMC5228201

[65] A. Condon , L. Graff , L. Elliot , and A. Ilnyckyj , “Acceptance of Colonoscopy Requires More Than Test Tolerance,” Canadian Journal of Gastroenterology 22, no. 8 (2008): 41–47. 18209780 10.1155/2008/107467 PMC2659119

[66] R. Melzack and P. D. Wall , “Pain Mechanisms: A New Theory,” Science 150, no. 3699 (1965): 971–979. 5320816 10.1126/science.150.3699.971

[67] L. M. Mendell , “Constructing and Deconstructing the Gate Theory of Pain,” Pain 155, no. 2 (2014): 210–216. 24334188 10.1016/j.pain.2013.12.010 PMC4009371

