# Supplementary material for: Effects of Virtual Reality on Anxiety, Stress, Pain, and Patient Satisfaction Among Palestinian Patients Undergoing Colonoscopy: Randomized Controlled Trial
Source: Health Sci Rep. 2026 Apr 27;9(5):e72420. doi: 10.1002/hsr2.72420 (PMC13121857; doi:10.1002/hsr2.72420)
Supplement: Supplementary file 1 — Supporting File 1 [file HSR2-9-e72420-s002.docx]

# CONSORT 2010 Checklist and Participant Flow Diagram

## Part A: CONSORT 2010 Checklist

Table S1.1 presents the completed CONSORT 2010 checklist for parallel-group randomized trials.

**Table S1.1. Completed CONSORT 2010 Checklist**

| **Section/Topic** | **Item** | **Checklist Item** | **Reported Where** |
| --- | --- | --- | --- |
| Title and Abstract | 1a | Identification as a randomized trial in the title | Title page |
|  | 1b | Structured summary of trial design, methods, results, and conclusions | Abstract |
| Introduction | 2a | Scientific background and explanation of rationale | Introduction |
|  | 2b | Specific objectives or hypotheses | Introduction (final paragraph) |
| Methods: Trial Design | 3a | Description of trial design (such as parallel, factorial) including allocation ratio | Methods: Study Design |
|  | 3b | Important changes to methods after trial commencement (such as eligibility criteria), with reasons | N/A — no changes made |
| Methods: Participants | 4a | Eligibility criteria for participants | Methods: Participants; Supplementary S2 |
|  | 4b | Settings and locations where the data were collected | Methods: Study Design |
| Methods: Interventions | 5 | The interventions for each group with sufficient details to allow replication, including how and when they were actually administered | Methods: Interventions; Supplementary S4 |
| Methods: Outcomes | 6a | Completely defined pre-specified primary and secondary outcome measures, including how and when they were assessed | Methods: Outcomes |
|  | 6b | Any changes to trial outcomes after the trial commenced, with reasons | N/A — no changes made |
| Methods: Sample Size | 7a | How sample size was determined | Methods: Sample Size |
|  | 7b | When applicable, explanation of any interim analyses and stopping guidelines | N/A |
| Methods: Randomization — Sequence Generation | 8a | Method used to generate the random allocation sequence | Methods: Randomization and Blinding |
|  | 8b | Type of randomization; details of any restriction | Methods: Randomization and Blinding |
| Methods: Allocation Concealment | 9 | Mechanism used to implement the random allocation sequence, describing any steps taken to conceal the sequence until interventions were assigned | Methods: Randomization and Blinding |
| Methods: Implementation | 10 | Who generated the random allocation sequence, who enrolled participants, and who assigned participants to interventions | Methods: Randomization and Blinding |
| Methods: Blinding | 11a | If done, who was blinded after assignment to interventions (for example, participants, care providers, those assessing outcomes) and how | Methods: Randomization and Blinding |
|  | 11b | If relevant, description of the similarity of interventions | Methods: Interventions |
| Methods: Statistical Methods | 12a | Statistical methods used to compare groups for primary and secondary outcomes | Methods: Statistical Analysis |
|  | 12b | Methods for additional analyses, such as subgroup analyses and adjusted analyses | Methods: Statistical Analysis |
| Results: Participant Flow | 13a | For each group, the numbers of participants who were randomly assigned, received intended treatment, and were analyzed for the primary outcome | Results; Supplementary S1 (Flow Diagram) |
|  | 13b | For each group, losses and exclusions after randomization, together with reasons | Results; Supplementary S1 (Flow Diagram) |
| Results: Recruitment | 14a | Dates defining the periods of recruitment and follow-up | Methods: Study Design |
|  | 14b | Why the trial ended or was stopped | N/A — completed as planned |
| Results: Baseline Data | 15 | A table showing baseline demographic and clinical characteristics for each group | Table 1 |
| Results: Numbers Analyzed | 16 | For each group, number of participants included in each analysis and whether the analysis was by original assigned groups | Results: Patient Flow |
| Results: Outcomes and Estimation | 17a | For each primary and secondary outcome, results for each group, and the estimated effect size and its precision | Tables 2–4 |
|  | 17b | For binary outcomes, presentation of both absolute and relative effect sizes is recommended | Tables 2, 4 |
| Results: Ancillary Analyses | 18 | Results of any other analyses performed, including subgroup analyses and adjusted analyses, distinguishing pre-specified from exploratory | Methods: Statistical Analysis |
| Results: Harms | 19 | All important harms or unintended effects in each group | Table 4; Results: Adverse Events |
| Discussion: Limitations | 20 | Trial limitations, addressing sources of potential bias, imprecision, and, if relevant, multiplicity of analyses | Discussion: Limitations |
| Discussion: Generalizability | 21 | Generalizability (external validity, applicability) of the trial findings | Discussion: Limitations |
| Discussion: Interpretation | 22 | Interpretation consistent with results, balancing benefits and harms, and considering other relevant evidence | Discussion |
| Other: Registration | 23 | Registration number and name of trial registry | Title page; Methods: Study Design |
| Other: Protocol | 24 | Where the full trial protocol can be accessed, if available | https://clinicaltrials.gov/study/NCT07145203 |
| Other: Funding | 25 | Sources of funding and other support (such as supply of drugs), role of funders | Declarations: Funding |

## Part B: CONSORT Participant Flow Diagram — Narrative Description

The flow of participants through the trial is described below and should be read alongside the CONSORT flow diagram figure submitted with the main manuscript.

**ENROLMENT**

Assessed for eligibility: n = 500

Excluded (n = 350):

- Did not meet inclusion criteria (n = 280):
  - Age >75 years (n = 85)
  - Previous colonoscopy (n = 112)
  - STAI score ≤38 (n = 58)
  - Medical contraindications (n = 25)
- Declined to participate (n = 70):
  - Preference for standard care only (n = 32)
  - Concerns about VR technology (n = 18)
  - Time constraints (n = 12)
  - No specific reason given (n = 8)

Randomized: n = 150

**ALLOCATION**

Allocated to VR group (n = 75): All received allocated intervention (n = 75)

Allocated to control group (n = 75): All received allocated intervention (n = 75)

**FOLLOW-UP**

Lost to follow-up: 0 in each group

Discontinued intervention: 0 in each group

**ANALYSIS**

Analyzed (VR group): n = 75 (intention-to-treat)

Analyzed (Control group): n = 75 (intention-to-treat)

Excluded from analysis: 0 in each group
