# Supplementary material for: Effects of Virtual Reality on Anxiety, Stress, Pain, and Patient Satisfaction Among Palestinian Patients Undergoing Colonoscopy: Randomized Controlled Trial
Source: Health Sci Rep. 2026 Apr 27;9(5):e72420. doi: 10.1002/hsr2.72420 (PMC13121857; doi:10.1002/hsr2.72420)
Supplement: Supplementary file 2 — Supporting File 2 [file HSR2-9-e72420-s001.docx]

# Full Eligibility Criteria

This supplementary material provides complete inclusion and exclusion criteria for participant enrolment, as summarized in the main manuscript.

## Inclusion Criteria

Participants were required to meet all of the following criteria:

1. Age between 18 and 75 years, to ensure adequate cognitive capacity without age-related contraindications to VR headset use.
2. Scheduled for elective diagnostic or surveillance colonoscopy (first-time colonoscopy only), to equalize expectation effects from prior procedural experience.
3. State-Trait Anxiety Inventory (STAI) State subscale score >38 at baseline screening, corresponding to moderate-to-severe anxiety, to target the population most likely to benefit from an anxiolytic intervention.
4. Ability to read and comprehend questionnaires written in Arabic language.
5. No cognitive impairment that would prevent the provision of valid informed consent or meaningful engagement with the VR equipment, as determined by clinical assessment.
6. Willingness to be randomized to either study group and to comply with all study procedures.

## Exclusion Criteria

Participants were excluded if any of the following applied:

### Clinical and Psychiatric Exclusions

1. Diagnosed major psychiatric disorder, including psychotic disorder, severe anxiety disorder (e.g., panic disorder with active episodes), or bipolar disorder, which could interfere with VR exposure or the accuracy of psychological outcome measures.
2. History of epilepsy or seizure disorder, given the theoretical risk of VR-induced photosensitive responses.
3. Uncontrolled hypertension, defined as systolic blood pressure >160 mmHg or diastolic blood pressure >100 mmHg at the pre-procedure assessment.
4. Chronic use of analgesic medications (opioids, gabapentinoids, or other centrally-acting pain modulators) that could confound procedural pain assessments.
5. American Society of Anesthesiologists (ASA) physical status classification of III or higher, reflecting severe systemic disease associated with elevated procedural risk.

### Functional and Sensory Exclusions

1. Visual acuity worse than 20/200 that cannot be corrected to within an acceptable range for VR headset use.
2. Inability to wear the VR headset due to corrective lenses that are incompatible with the headset form factor and cannot be otherwise accommodated.
3. Cognitive impairment preventing valid consent or reliable questionnaire completion, as assessed by clinical judgment.
4. Inability to communicate verbally or respond to questionnaires (e.g., profound hearing impairment without adequate accommodation, mutism).

### Device-Specific Exclusions

1. Presence of implanted electronic devices (e.g., cochlear implants, certain cardiac pacemakers or implantable cardioverter-defibrillators) for which VR headset use is contraindicated per manufacturer guidelines.

### Methodological Exclusions

1. Receipt of any anxiolytic, sedative, or hypnotic medication within 24 hours prior to the procedure, to avoid confounding of baseline and post-procedural anxiety and pain assessments.
2. Intraoperative complications requiring early termination of the colonoscopy or emergency medical intervention.
3. Presentation for emergency (non-elective) colonoscopy, as this context does not allow for baseline assessment or standardized VR setup.

**Note:** All eligibility criteria were assessed by a trained research nurse immediately after the patient was scheduled and prior to enrolment. Eligibility confirmation was re-verified on the day of the procedure before randomization and baseline data collection.
