# Supplementary material for: Effects of Virtual Reality on Anxiety, Stress, Pain, and Patient Satisfaction Among Palestinian Patients Undergoing Colonoscopy: Randomized Controlled Trial
Source: Health Sci Rep. 2026 Apr 27;9(5):e72420. doi: 10.1002/hsr2.72420 (PMC13121857; doi:10.1002/hsr2.72420)
Supplement: Supplementary file 3 — Supporting File 3 [file HSR2-9-e72420-s004.docx]

# VR Cultural Adaptation Protocol

This supplementary file describes the comprehensive, multi-phase process used to culturally adapt the virtual reality (VR) intervention for Palestinian patients. The adaptation followed recognized frameworks for cultural competence in healthcare interventions [Refs 34,35 in main manuscript] and was developed collaboratively with multiple stakeholder groups.

## Phase 1: Needs Assessment and Stakeholder Engagement

A structured needs assessment was conducted prior to content development. This involved:

- Demographic and cultural profiling of the target population served by Rafidia Surgical Governmental Hospital, including primary language, religious practices, gender-specific healthcare preferences, and digital technology literacy.
- Semi-structured interviews with five experienced endoscopy nurses and two gastroenterologists to identify patient concerns and common emotional reactions to colonoscopy.
- Consultation with a Palestinian Cultural Advisory Board comprising two community leaders, two patient advocates, and three primary care physicians familiar with local health-seeking behaviors.
- Review of published literature on cultural influences on procedural anxiety in Arab and Palestinian populations [Refs 52–54 in main manuscript].

## Phase 2: Linguistic Adaptation

All guided audio meditation content was produced professionally in Palestinian colloquial Arabic, rather than standardized Modern Standard Arabic or literal English translation. Specific adaptations included:

- Use of locally recognizable idiomatic expressions, proverbs, and descriptive language familiar to a West Bank Palestinian audience.
- Incorporation of culturally resonant metaphors, including references to the olive harvest season, seasonal agricultural cycles, limestone terrain, and coastal landscapes associated with Palestinian cultural memory.
- Provision of gender-specific narration options: participants could select either a male or female narrator voice, according to personal preference and cultural comfort.
- Calibration of narration pace and tone to reflect a calm, reassuring, and culturally appropriate communication style, avoiding overly clinical or formal register.

## Phase 3: Visual Content Customization

The VR visual environments were selected and modified to include imagery with relevance to Palestinian geography and cultural identity:

- Mediterranean coastal scenes evoking Palestinian seashores and associated leisure and family experiences.
- Olive groves and terraced agricultural fields characteristic of traditional Palestinian farming regions and national cultural identity.
- Natural and semi-urban scenes featuring limestone architecture common in Palestinian towns and villages.
- Seasonal imagery was synchronized with the local Palestinian climate and agricultural calendar (autumn–winter harvest) to enhance authenticity and relatability.

## Phase 4: Integration of Islamic Meditation Principles

Given that the majority of the Palestinian population identifies as Muslim, the guided meditation content was developed to integrate concepts consistent with Islamic mindfulness and spiritual reflection traditions, without religious prescription. Key elements included:

- Subtle references to Islamic contemplative practices: muraqabah (attentive self-awareness) and tafakkur (purposeful reflection on creation and the natural world) were woven into narration without doctrinal framing.
- Breathing exercises structured to synchronize with calm, rhythmic patterns inspired by traditional Islamic meditative cadence.
- Affirmations and reassurance phrases drawing on Islamic conceptions of patience (sabr), trust (tawakkul), and gratitude (shukr).
- Careful exclusion of any imagery, sounds, or language inconsistent with Islamic values, including music with instruments considered objectionable; only nature soundscapes (water, wind, birdsong) were used as background audio.
- Optional inclusion of quiet Quranic recitation at low volume as an alternative ambient audio track, available on patient request.

## Phase 5: Multi-Level Cultural and Religious Review

Prior to pilot testing, all adapted content underwent a structured review process:

1. Islamic scholar review: Two local Islamic scholars (ulama) reviewed all narration scripts, visual descriptions, and audio components to confirm consistency with Islamic teachings and absence of problematic content.
2. Cultural Advisory Board review: The Palestinian Cultural Advisory Board (Phase 1) reviewed full content for cultural authenticity, relevance, and acceptability.
3. Mental health professional review: Two clinical psychologists with experience in culturally responsive care for Palestinian patients reviewed meditation scripts for psychological safety and cultural appropriateness.
4. Clinical staff review: Three endoscopy nurses reviewed the intervention for practical feasibility, patient comfort during the procedure, and potential contraindications.

All reviewer feedback was documented, discussed by the research team, and incorporated through iterative revision cycles.

## Phase 6: Pilot Testing and Refinement

Prior to the main RCT, the adapted VR program was pilot tested with 20 Palestinian adults (10 male, 10 female) not enrolled in the main trial. Pilot objectives included:

- Assessment of content acceptability, cultural resonance, and comfort.
- Evaluation of VR system usability and headset tolerability.
- Identification of any adverse reactions (dizziness, disorientation, discomfort).
- Feedback on session duration, audio volume, and environmental scene options.

Results of pilot testing:

- 95% of pilot participants (n=19/20) rated the VR content as culturally appropriate and comfortable.
- No serious adverse effects were reported; two participants noted mild initial disorientation that resolved within 2 minutes.

Refinements implemented following pilot feedback:

- Audio volume range expanded and made individually adjustable.
- A fourth environmental scene (Snowy Landscape) added to provide greater choice for patients with varied preferences.
- The introductory orientation sequence (first 2–3 minutes of each scene) modified to slow environmental transitions and reduce initial disorientation.
- Narration pacing slowed by approximately 10% following feedback that the original pace felt rushed for a relaxation context.

## Summary of Final VR Content

The final culturally adapted VR program included the following four immersive environments:

| **Scene** | **Visual Description** | **Audio Components** |
| --- | --- | --- |
| Tropical Beach | Sandy Palestinian-style beach, warm sunlight, palm trees, gentle surf and seagulls | Guided meditation (Arabic), wave sounds, nature ambience |
| Forest Walk | Dappled sunlit forest walkway, soft breeze, woodland wildlife | Guided meditation (Arabic), birdsong, gentle wind |
| Underwater Diving | Calm Mediterranean underwater scene, coral, colourful fish, soft currents | Guided meditation (Arabic), underwater ambient sounds |
| Snowy Landscape | Snow-capped mountains, evergreen trees, quiet stillness | Guided meditation (Arabic), soft wind, silence |

All scenes included: (1) 15–30 minute Palestinian Arabic guided meditation narration; (2) culturally appropriate nature sounds; (3) breathing exercises integrated with Islamic meditative patterns; (4) progressive relaxation guidance; and (5) culturally contextualized affirmations.
