# Supplementary material for: Effects of Virtual Reality on Anxiety, Stress, Pain, and Patient Satisfaction Among Palestinian Patients Undergoing Colonoscopy: Randomized Controlled Trial
Source: Health Sci Rep. 2026 Apr 27;9(5):e72420. doi: 10.1002/hsr2.72420 (PMC13121857; doi:10.1002/hsr2.72420)
Supplement: Supplementary file 4 — Supporting File 4 [file HSR2-9-e72420-s003.docx]

# Standardized Intervention and Control Group Protocols

This supplementary file provides full standardized protocols for both the VR intervention group and the control group, enabling replication of the study procedures.

## Part A: VR Group Protocol

### A1. Equipment

VR headset: Meta Quest 2 (Meta Platforms, Inc.) — standalone, wireless; 1832×1920 px resolution per eye; ~90° field of view; built-in spatial audio; adjustable, padded head straps.

Software: Guided Meditation VR application, culturally adapted for Palestinian patients (see Supplementary Material S3). Four pre-loaded immersive nature environments available for patient selection.

Infection control: All headset surfaces (facial interface, head straps, outer casing) disinfected between each patient use with hospital-approved disinfectant wipes per manufacturer guidelines and institutional infection control policy.

### A2. Staff Training

All research nurses and study assistants involved in VR administration completed a standardized two-hour training program covering:

- VR device operation, navigation, and troubleshooting.
- Infection control and headset cleaning procedures.
- Patient education and orientation techniques for first-time VR users.
- Identification and management of VR-related adverse effects (dizziness, nausea, disorientation).
- Documentation of VR side effects using the adapted Simulator Sickness Questionnaire.
- Cultural sensitivity in assisting patients with environment selection.
- Discontinuation criteria and emergency response procedures.

### A3. Pre-Procedure Setup (10 minutes before colonoscopy)

1. Introduce the patient to the VR technology: explain the purpose, how the headset works, and how to communicate any discomfort during the procedure (standardised verbal script used).
2. Clean and inspect the headset per infection control protocol.
3. Fit the headset on the patient while they are in a seated or semi-recumbent position; adjust head straps for comfort and secure fit.
4. Provide a brief 2–3 minute orientation preview of each available scene. Allow the patient to view and select their preferred environment.
5. Confirm audio volume is comfortable (patient-adjusted within a pre-set range of 40–70 dB).
6. Remove the headset temporarily while the patient is positioned for the procedure (left lateral decubitus) and while IV access and monitoring are established.

### A4. During the Procedure

1. Re-seat and secure the VR headset when the patient is in the procedure position and the gastroenterologist is ready to begin scope insertion.
2. Launch the patient's pre-selected VR environment. Begin the guided meditation audio.
3. Confirm the patient's comfort (audio volume, headset fit) immediately after launch.
4. The VR intervention is passive: patients observe the pre-selected immersive virtual environment. No handheld controllers are used during the colonoscopy.
5. A trained study assistant remains present in the room throughout the procedure to monitor for device-related adverse effects and patient distress.
6. Volume adjustments may be made by the assistant upon patient request.
7. The VR exposure is maintained continuously for the duration of the colonoscopy (typically 15–30 minutes).

### A5. Discontinuation Criteria

The VR intervention shall be paused or permanently discontinued if the patient:

- Reports nausea or vomiting.
- Reports severe dizziness or spatial disorientation that does not resolve within 2 minutes of repositioning.
- Requests removal of the headset for any reason.
- Requires emergency medical intervention or procedural complications arise requiring urgent gastroenterologist attention.
- Exhibits signs of extreme distress, agitation, or dissociation.

All discontinuations shall be documented with reason and time noted.

### A6. Post-Procedure

1. Remove the headset immediately upon completion of scope withdrawal.
2. Conduct a brief (2–3 minute) standardized check-in to ask about the VR experience and any discomfort.
3. Administer the adapted Simulator Sickness Questionnaire (4-point severity scale: 0=none, 1=mild/no intervention required, 2=moderate/break needed, 3=severe/discontinuation required).
4. Reassess the patient at 15 minutes post-procedure for any delayed adverse effects.
5. Clean and disinfect the headset per protocol and store for next use.

## Part B: Control Group Protocol

### B1. Pharmacological Sedation

Both groups received conscious sedation per standard hospital protocol:

- Midazolam: initial dose 1–3 mg IV, titrated based on patient age, weight, and clinical response; repeat doses administered per gastroenterologist discretion.
- Fentanyl: initial dose 25–50 mcg IV, adjusted based on patient comfort and vital signs; supplemental doses administered at gastroenterologist discretion.

Sedation decisions were made entirely by the performing gastroenterologist according to clinical judgment, independent of the research protocol.

### B2. Environmental Standardization

The following environmental conditions were maintained identically across all control group procedures and all VR group procedures to ensure that environmental factors did not confound between-group comparisons:

- Ambient room temperature: 22–24°C.
- Lighting: standard fluorescent procedure room lighting; identical across all procedures.
- Background noise: limited to sounds from medical equipment only; no music, television, or other auditory stimulation.
- Room configuration and equipment placement: standardized for all procedures.
- Family members and visitors: not permitted in the procedure room, per institutional infection control policy.

### B3. Standard Care Procedures

All control group patients received the following standard perioperative care:

- Standard pre-procedure explanation of the colonoscopy process using the hospital's routine patient information script.
- Routine communication and verbal support during the procedure, following standard clinical protocol; no additional verbal reassurance, distraction coaching, or psychological support was provided.
- No television, music, guided imagery, progressive muscle relaxation, or any other non-pharmacological distraction or anxiety-reduction technique was employed.
- Continuous hemodynamic monitoring (HR, SBP, DBP, SpO₂) throughout the procedure.
- Post-procedure recovery care per standard institutional protocol.

## Part C: Data Collection Protocol

### C1. Assessor Training

Four research nurses completed a two-day training program covering: standardized questionnaire administration (STAI, PSS-10, VAS, NRS, satisfaction VAS); timing and sequence of data collection; procedures to maintain assessor blinding; hemodynamic parameter measurement and recording; and adverse event documentation. Inter-rater reliability was confirmed through practice assessments (intraclass correlation coefficient >0.90 for all measures involving assessor judgment).

### C2. Data Collection Timeline

| **Time Point** | **Timing** | **Variables Collected** |
| --- | --- | --- |
| T0 — Baseline | 30–60 min before procedure; after consent | STAI State subscale; SBP, DBP, HR; demographic and clinical data |
| Intra-operative | 15, 30, and 45 min after scope insertion (where applicable) | HR, SBP, DBP (recorded from continuous monitoring by blinded nurse post-procedure) |
| Immediate post-procedure | Within 5 min of scope withdrawal | Pain VAS (0–10); Pain NRS (0–10) |
| T1 — Recovery | 30 min after procedure completion | STAI State subscale (post); PSS-10; Satisfaction VAS (0–100); post-procedure HR, SBP, DBP |
| VR AE Assessment | Immediately post-VR and at 15 min post-procedure (VR group only) | Adapted Simulator Sickness Questionnaire |

### C3. Blinding Procedures During Data Collection

Research nurses collecting outcome data were fully blinded to group allocation. They collected all data in a separate area from the procedure room and had no access to randomization records or VR setup equipment. Hemodynamic data recorded by monitoring equipment during the procedure were reviewed and transcribed by blinded research nurses after each procedure using de-identified monitoring printouts. Statistical analysis was conducted with groups coded as 'A' and 'B' until all primary analyses were complete.
